# Confident Expectation in the Cure of Disease

**Published:** 1846-02

**Authors:** 


*Confident Expectation in the Cure of Disease.*—Dr. Paris mentions a curious example of the salutary effect of confident expectations in the relief of disease. When the powers of the nitrous oxide were discovered, Dr. Beddoes at once concluded that it must be a specific for paralysis: a patient was selected for the trial, and the management was entrusted to (Sir Humphry) Davy. Previous to the administration of the gas, the young chemist inserted a small pocket thermometer under the tongue of the patient, as he was accustomed to do upon such occasions, to ascertain the degree of animal temperature, with a view to future comparison. The paralytic man, wholly ignorant of the nature of the process to which he was to submit, but deeply impressed, from the representations of Dr. Beddoes, with the certainty of its success, no sooner felt the thermometer under his tongue, than he concluded the *talisman* was in full operation, and in a burst of enthusiasm declared that he already experienced the effect of its benign influence throughout his whole body: the opportunity was too tempting to be lost; Davy cast an intelligent look at Mr. Coleridge, and desired his patient to renew his visit on the following day, when the same ceremony was performed, and repeated every succeeding day for a fortnight, the patient gradually improving during that period, when he was dismissed as cured, no other application having been used.—*Med. Chir. Rev.—Ibid.*

